# Histone acetylation functions in the wound-induced spore formation in nori

**DOI:** 10.3389/fpls.2022.1064300

**Published:** 2022-12-08

**Authors:** Xiaowei Guan, Huijuan Qian, Weihua Qu, Shanshan Shu, Ying Pang, Nianci Chen, Xiaoqian Zhang, Yunxiang Mao, Ansgar Poestch, Dongmei Wang

**Affiliations:** ^1^ Key Laboratory of Marine Genetics and Breeding Ocean University of China (OUC), Ministry of Education, Qingdao, China; ^2^ College of Marine Life Sciences, Ocean University of China, Qingdao, China; ^3^ Key Laboratory of Utilization and Conservation for Tropical Marine Bioresources, Ministry of Education, Hainan Tropical Ocean University, Sanya, China

**Keywords:** *Nori*, *Pyropia yezoensis*, wound-induced spores, histone deacetylase, SAHA, ROS

## Abstract

The red macroalgae *Pyropia yezoensis* is one of the most economically important marine crops. In the asexual reproduction process, released archeospores could provide secondary seedling resources in nori farming and be used to establish asexual seeding strategies. We previously found that wounds could induce the somatic cells in sectioned *Pyropia* thalli to develop into large number of asexual wound-induced spores (WIS) in a short time. Many genes involved in signaling pathways, cell division, cell wall remodeling, etc. exhibited transcriptional variation in this cell fate transition process. However, the regulatory mechanisms controlling gene transcription remain elusive. In this study, we found that suberoylanilide hydroxamic acid (SAHA), the inhibitor of histone deacetylase, strongly repressed WIS formation after wounding. The lack of a sharp increase in HDAC activity after wounding, as well as the hyperacetylated status of histone H3 and H4, were observed in SAHA-treated thalli fragments, thus confirming a histone deacetylation-related epigenetic mechanism of wound-induced cell fate reprogramming. Moreover, histone deacetylation is required in the whole process of WIS formation and release. We further compared the genome-wide transcriptional variations after SAHA treatment. SAHA-responsive genes were identified, including some transcriptional factors, chromatin remodeling complex proteins, protein kinases, etc. Transcription of RBOH genes was also altered by SAHA, and moreover, ROS signals in cut fragments were attenuated, both indicating that the ROS systematic signaling pathway is closely associated with histone deacetylation. Our findings provide insights into the biological significance of dynamic histone acetylation states in WIS formation in *P. yezoensis*.

## 1 Introduction

*Pyropia yezoensis* (also named as *Neopyropia yezoensis* (Yang et al., 2020)), Bangiales, is a red seaweed with important economic value (Blouin et al., 2011). Thalli, the gametophyte stage, contains valuable nutrients and is the material for nori. In the present marine cultivation of *P. yezoensis*, the gametophytes seedlings were generated from the germination of conchospores. The discharge of conchospores relies on the pond cultivation of sporophyte conchocelis in mollusk shells for 4–5 months. An alternative method to alleviate the time and cost-consuming strategy is the asexual reproduction of *P. yezoensis*. Under certain environmental conditions, vegetative cells along the margin of young thalli usually dedifferentiate into asexual archeospores, which would grow into individual healthy thalli after release and attachment onto fibrous nets (Mei et al., 2001; Sahoo et al., 2002). Archeospores have been proven to be an important secondary source of seedlings in marine cultivation. However, synchronous and efficient production of archeospores is difficult to achieve due to the fluctuating environmental changes in the ocean, which strongly limits the application of naturally released archeospores as the primary seedling source. Previously, researchers found that cutting thallus into small fragments could induce the intensive formation of “spores” within one week (Chen et al., 2020). This inexpensive and easy-operating method was a promising way to produce both primary and secondary seedlings. Further improving the productivity of “spores” requires a deep understanding of the underlying biological mechanisms.

From the perspective of cell biology, the formation of “spores” is a process of wound-induced cell fate transition from vegetative cells to “spores,” or called cell reprogramming. According to morphological observation and gene expression analysis, it can be divided into three phases, including systematic transduction of the wound signals, cell proliferation, and spore maturation, in each of which many genes (including those related to ROS and Calcium signaling pathways, cell division, cell wall remodeling etc.) exhibit sequential transcriptional changes at certain time points (Guan et al., 2022). However, the regulatory mechanisms that coordinate the gene expression remain unknown.

A wealth of studies showed that chemical modifications on the lysine residues of histones, e.g., methylation, acetylation, could change the chromatin conformation to open or close the transcription of target genes and play essential roles in cellular development, stress response, etc. in animals and plants. Histone acetylation, such as H3K9/14ac and H3K27ac, orchestrates the wound-induced transcriptional activation and pluripotency acquisition in land plants and aquatic moss (Rymen et al., 2019). The balance of histone acetylation is modulated by the coordinated actions of histone acetyltransferase (HAT) and histone deacetylase (HDAC). Interfering the enzymatic activity of HDACs by specific inhibitors, such as SAHA or trichostatin A (TSA), or gene mutation, resulted in repressed or impaired callus formation at wound sites, along with an increased global H3 and H4 acetylation levels (Li et al., 2014; Lee et al., 2016). Accordingly, mutations in HAT genes also cause severe defects in callus formation or tissue regeneration after wounding (Kim et al., 2018).

Despite the importance of epigenetic regulation in cell fate reprogramming in both animals and land plants, the biological relevance of histone acetylation in red algae is still elusive, except several genes encoding the SWI chromatin remodeling factors, which are the “sensors” of histone modification status, were identified in *Pyropia* genomes (Stiller et al., 2018). The wound-induced spore formation in *P. yezoensis*, combining the availability of genome information, makes *P. yezoensis* an ideal research model of cell reprogramming in red algae (Guan et al., 2022). In this study, we investigated the effect of SAHA on wound-induced spore formation in *P. yezoensis* and revealed that HDACs act as an essential epigenetic switch that renders the cell reprogramming of vegetative cells by modulating transcriptional levels of candidate target genes.

## 2 Materials and methods

### 2.1 *Pyropia* culture and SAHA treatment

*Pyropia yezoensis* pure line RZ was used in this study. Thalli were cultured in Provasoli Enriched Seawater (PES) medium (Provasoli, 1968) under 50 μmol photons m^-2^s^-1^ with a 12h light/12h dark photoperiod. Cutting thalli into fragments was performed exactly as described by Guan et al. (Guan et al., 2022). SAHA (APExBIO, A4084) was dissolved in DMSO to prepare a 1.0 M stock solution. An appropriate volume of the stock was added to PES media to obtain SAHA-treated media with a final concentration of 0.1 mM. The same amount of DMSO was added as a control medium. For SAHA treatment, cut fragments were put into SAHA-treated media immediately after wounding. For microscopic observation and calculating the mortality and spore-releasing probability, each fragment was put into one well of a 96-well plate. Every 30 fragments were one biological replicate and three replicates were prepared for each treatment. P-values were estimated by means of the t-test method (n=3). For SAHA pre-treatment, fragments in SAHA-treated media were transferred into control media at the 1, 2, 3, 4, and 5 days after wounding. For SAHA post-treatment, fragments in control media were transferred into SAHA-treated media at corresponding time points. To collect samples for transcriptomic analysis and immunoblotting analysis, cut fragments were cultured in conical flasks containing control media or SAHA-treated media respectively, and collected at different time points after wounding depending on experimental requirements (three repetitions at each time point).

### 2.2 HDAC enzymatic activity assay

Wounded thallus fragments in control and SAHA-treated media were collected at the 6^th^, 24^th^ and 48^th^ hour after wounding, respectively, each with three biological replicates. Nucleoprotein was extracted using the Nuclear and Cytoplasmic Protein Extraction Kit (Beyotime) according to the manufacturer’s instructions. Concentrations of the extracted nucleoprotein were determined by the BCA Protein Assay Kit (Beyotime). Histone deacetylase activity was detected with Epigenase™ HDAC Activity/Inhibition Direct Assay Kit (EPIGENTEK).

### 2.3 Transcriptomic data analysis

Three replicates of SAHA-treated fragments were sampled at the 6th hour, 1, 2, 3, and 5 days after wounding by collection and centrifugation. Triplicates of “uncut thalli” were collected as controls. RNA isolation, mRNA-Seq library preparation were done with NEBNext^®^ UltraTM RNA Library Prep Kit (NEB, America) following the manufacture’s instruction. PE 2×100 bp sequencing was performed on Illumina HiSeq2000 platform for each sample. After filtering out low quality reads, adapter sequences and reads containing N, the generated clean data were aligned to the assembly genome using HISAT2 (Kim et al., 2019). The mRNAs expression was calculated as Reads Per Kilobase per Million reads (RPKM). The differential expression of mRNAs was performed using the DESeq2 R package (1.10.1) (Love et al., 2014). The criterion of adjusted P-value <0.05 was used to identify differentially expressed genes.

### 2.4 ROS detection

DCFH-DA (Solarbio, D6470) was used to detect ROS of cut fragment control media, 0.1 mM SAHA-treated media and 0.5 μM TSA (APExBIO, A8183) treated media. Cut fragments were incubated in medium with 5-μM DCFH-DA for one hour, then washed twice with seawater. ROS signals were observed under a microscope (Nikon ECLIPSE 80i). The fluorescence was excited at 504 nm and detected at 529 nm.

### 2.5 Immunoblotting analysis

Nuclear proteins of each sample were extracted by sucrose density gradient centrifugation and quantified by BCA protein assay kit (P0010, Beyotime). Equal amount of nuclear protein samples were loaded for SDS-PAGE electrophoresis. After that, the proteins were transferred to a polyvinylidene difluoride membrane and blocked with 8% milk powder in TBST. Then the blotted membrane was incubated with primary antibody at room temperature for 2 h. The primary antibodies used in this study were as follows: anti-Acetyllysine Mouse mAb (1:1000; PTM-101; PTM BIO), and anti-histone H3 (1:2000; ab1791; Abcam). Secondary antibodies were HRP Goat Anti-Mouse IgG (H+L) (1:6000, AS003, ABclonal), and HRP Goat Anti-Rabbit IgG (H+L) (1:4000, AS014, ABclonal). The signals of them were detected using enhanced chemiluminescence (AR1190, BOSTER).

### 2.6 Data availability

The datasets presented in this study can be found in online repositories with Bioproject accession number as PRJNA888147 and the SRA accession numbers from SAMN31210159 to SAMN31210173.

## 3 Results

### 3.1 SAHA repressed the formation of wound-induced spores

**Figure 1:**
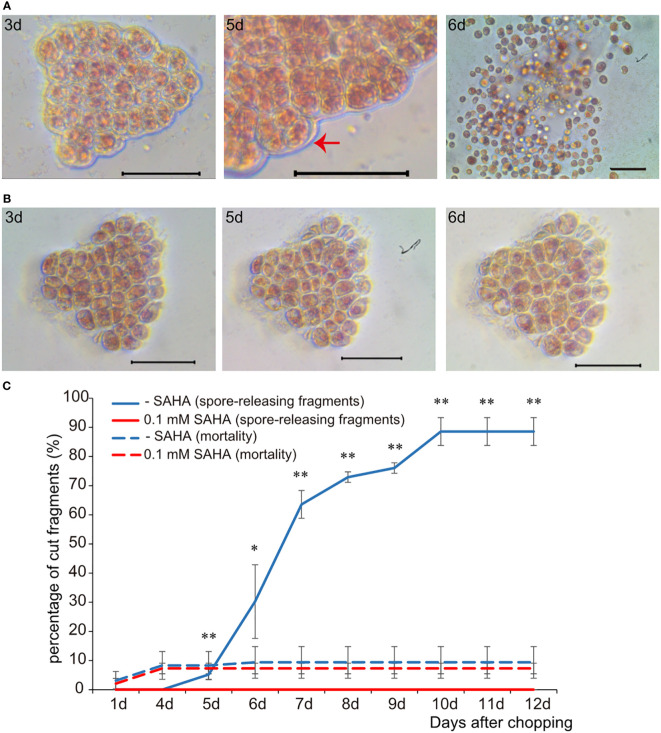
The effect of SAHA on the cellular development of wounded *P. yezoensis* thallus fragments. **(A)** Morphological changes of the same thallus fragment tracked at 3, 5, and 6 days after wounding. The image of day 5 was zoomed out to show multiple cellular sporangia. **(B)** Morphological changes of one SAHA-treated fragment tracked at the same time point after wounding. **(C)** Effects of SAHA on the percentage of cut fragments able to release spores. Average percentages of spore-releasing fragments plotted over time in control and SAHA-treated media; error bars represent the SD (n = 3, 30 fragments/replicate as described in “Materials and Methods” section). *, 0.01 < P-value < 0.05; **, P-value < 0.01.

In order to examine the role of histone acetylation in the epigenetic control of the wound-induced spore formation from thalli somatic cells, the effect of SAHA on the development of *P. yezoensis* cut fragments was assessed. Initial dosage experiments were used to establish the minimal concentration of SAHA (0.1 mM) in relation to the specific phenotypes discussed below (**Supplementary Figures S1A, B**). Cut fragments cultured in the control medium formed large multicellular sporangia on day 5 and started to release spores on day 6, as we observed previously (**Figure 1A**). When supplementing with SAHA from the onset of cutting, somatic cells rounded up, with dispersed chloroplast pigments and enlarged intercellular space. In contrast to the newly-formed thick wall around the fragments in control media, there was still surrounding cell debris left on day 3 and even day 5. Moreover, the average cell numbers in each fragment did not change and even dropped slightly due to occasional cell death (**Figure 1B**; **Supplementary Figure S2**), suggesting the lack of cell division events after wounding. Congruently, we did not observe any “packet”-like sporangial structure. Afterwards, these fragments were maintained as partial thalli, albeit lacking any morphological differentiation, such as rhizoids (**Figure 1B**). The death rate of fragments under either condition was comparable, lower than 15%, mostly happened in the first four days. We counted the number of cut fragments that could release spores in the 96-well plates. The probability of releasing spores in the control medium was more than 70% on day 8 and reached 85% on day 9, while it was nearly zero in SAHA-treated condition (**Figure 1C**). Therefore, SAHA not only affected the edge recovery from wound stress but also strongly repressed the fate reprogramming from somatic cells to pluripotent spores. Similar repression was observed when treated with TSA, another inhibitor of HDAC (**Supplementary Figures S1C, D**). When treated with the chemical inhibitor of histone acetyltransferase, MB-3, WIS formation was not significantly affected. When the mortality of fragments reached to more than 10.0%, 95% of the left alive fragments could generate and release spores at the 8^th^ day after wounding (**Supplementary Figure S3**).

### 3.2 Increased HDAC enzymatic activity is required after wounding

**Figure 2:**
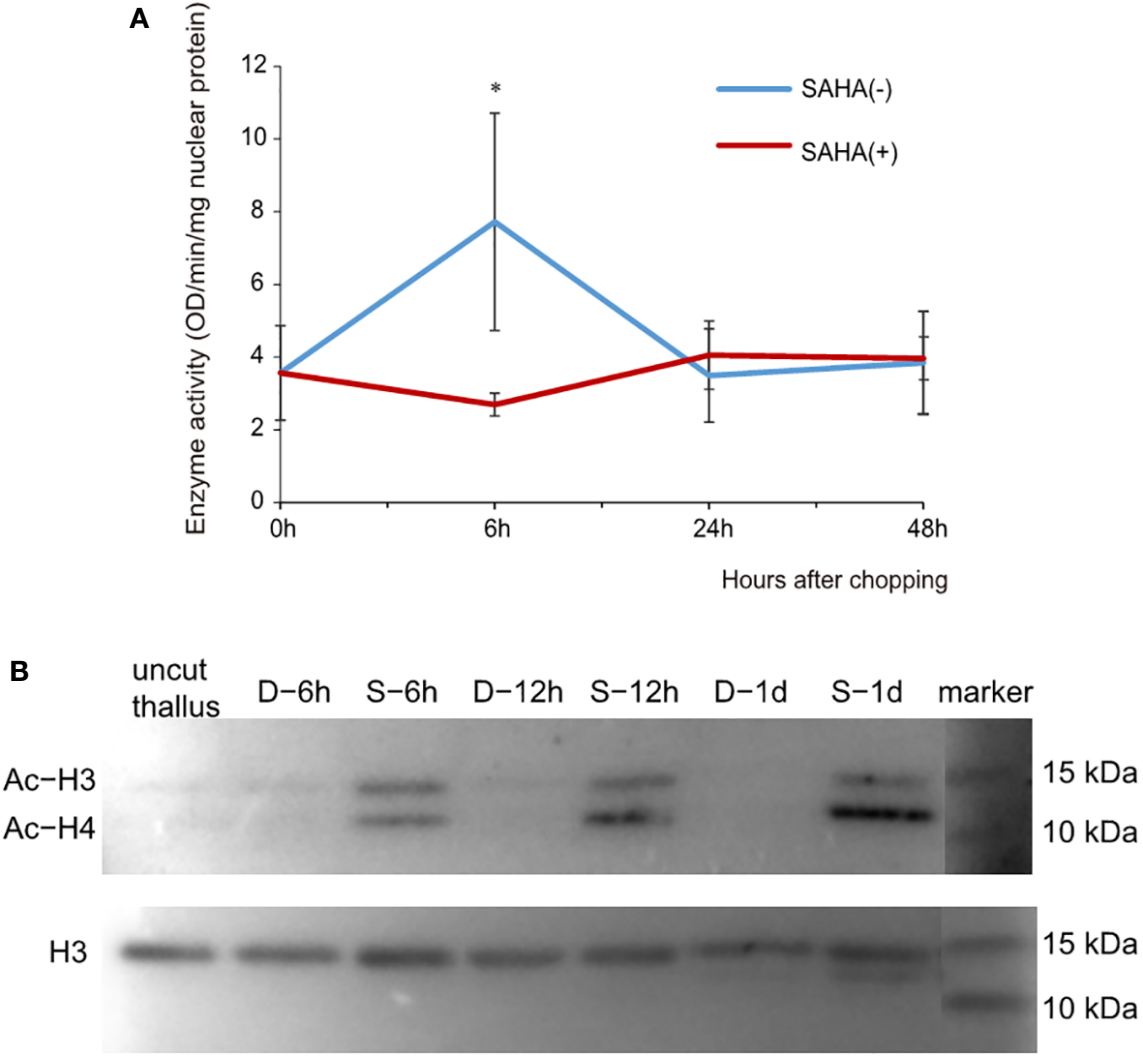
Effects of SAHA on the enzymatic activity of HDAC and acetylation levels of histone proteins in thallus fragments after wounding. **(A)** Changes of HDAC activities in cut fragments in control or SAHA-supplemented media. Error bars represent the SD (n = 3). *, 0.01 < P-value < 0.05. **(B)** Immunoblot of acetylated (Ac) histone H3 and H4 in cut fragments in control (marked as D-) or SAHA-supplemented media (marked as S-). Simultaneous staining of total histone H3 was used as internal control.

To verify the assumption that SAHA repressed the formation of wound-induced spores *via* a histone acetylation-related mechanism, we evaluated the activity of HDACs in the cut fragments cultured in media with or without SAHA (**Figure 2A**). The enzymatic activity of HDAC in thallus was 0.67 OD/min/mg nuclear protein. In cut fragments cultured in control medium, the activity increased more than two folds (1.47) at the 6^th^ hour and then dropped back to a level comparable to that of thallus at the 24^th^ hour. When cultured on the SAHA-supplemented media, the significant increase in HDAC activity was strongly repressed at 6^th^ hour, but maintained at a merely varied level after wounding. This analysis, combining the above observations of cellular development, suggested that SAHA treatment repressed the wound-induced spore (WIS) formation by restraining the early enhancement of HDAC activity which was required in mounting the wound-induced cell fate reprogramming.

We further determined the acetylation status of histone proteins in cut fragments in control or SAHA-treated media using a pan acetyl-lysine antibody. Histone H3 was simultaneously detected as internal control. We observed two clear bands of immunoblotting signals in the range of 10 to 25 KD, one corresponding to histone H3 and the other showing a similar size to H4 (**Figure 2B**). Compared to the uncut thalli, the histone acetylation level of H3 exhibited little change at 6h in cut fragments in control media and reduced to be barely detected at 24h. However, hyperacetylation of H3 was observed in the SAHA-treated fragments at 6h and maintained at 12h and 24h. Increased acetylation levels were also observed for histone H4, with even higher levels compared to H3 in the same sample (**Figure 2B**). These results suggest that SAHA induces hyperacetylation of histone H3 and H4 in cut fragments, which may lead to the block of cell fate transition.

### 3.3 Histone acetylation is involved in the whole process of wound-induced spore formation and release

**Figure 3:**
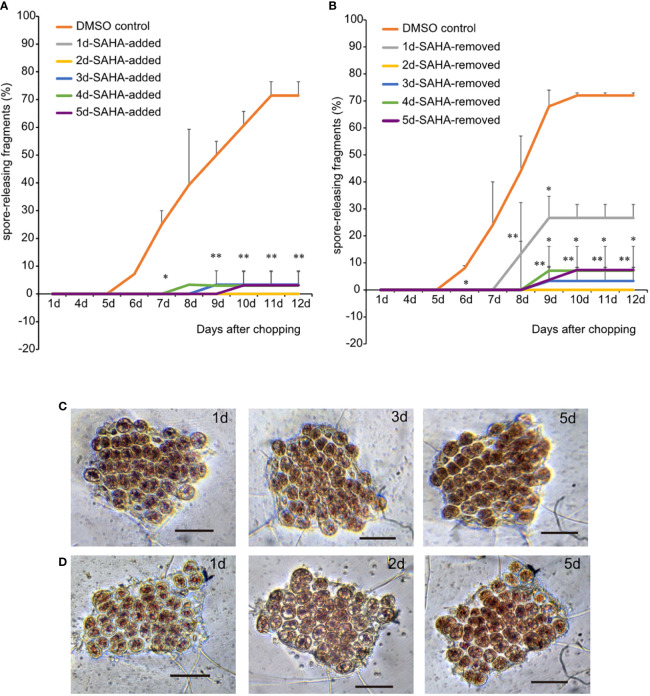
Effects of transient SAHA treatment on wound-induced spore formation. **(A)** Effect of SAHA post-treatment on the percentage of spore formation. The sample with SAHA added to media on day 1 after wounding was marked as 1d-SAHA-added and so as the other samples. Error bars represent the SD (n = 3, 30 fragments/replicate as described in “Materials and Methods” section).*, 0.01 < P-value <0.05; **, P-value < 0.01. **(B)** Effect of SAHA pre-treatment on the percentage of spore formation. The sample with SAHA removed on day 1 after wounding was marked as 1d-SAHA-removed and so as the other samples. **(C, D)**, The morphological changes of 1d-SAHA-added **(C)** and 2d-SAHA-added **(D)** thallus fragments (Bar = 50 μm).

The entire process of wound-induced spore formation takes 5–6 days and involves a series of cellular activities, including local healing at the wound sites, cell dedifferentiation accompanied by typical cell divisions, the formation of sporangia “packet”, maturation and releasement of pre-spores. In spite of the enhanced activity at the early stage of wound response, whether HDACs also play roles in later developmental stages? To address this question, we changed to SAHA-supplemented medium at 0 h, 1d, 2d, 3d, 4d and 5d after wounding, respectively. Fragments in control media started to release spores on day 6, and the percentages reached over 80% by day 12. However, nearly all the fragments in SAHA-supplemented media, no matter when SAHA was added, did not release any spores (**Figure 3A**). In 0h-SAHA-added and 1d-SAHA-added fragments, cells remained alive, albeit with dispersed chloroplast pigments and enlarged cell size. Cell divisions were not observed in them (**Figure 3C**). When SAHA was added on the 2^nd^ day after wounding, cell numbers apparently increased afterwards and the fragments became crowded, suggesting that cell divisions occurred at least once (**Figure 3D**). Active cell divisions were also observed in fragments when SAHA-treatment started on days 3, 4, or 5. In the 5d-SAHA-added fragments, although the “packet” structures of sporangia were already observed before SAHA addition, pre-spores were not released, but enclosed in the thick cell wall of sporangia. Ten days later, the “packet” structure of sporangia disappeared, and the intercellular space increased (**Supplementary Figure S4A**). However, we added SAHA to the fragment culture at the onset of wounding, but removed it by changing to control media on days 1, 2, 3, and 5, respectively. Among fragments that were treated by SAHA only on day 1, only a few fragments started to release spores on day 7, and the final probability of spore-formation as counted on day 12 reduced to 30%, compared with about 75% in control media. Fragments that were under the first two or more days of SAHA treatment barely formed typical sporangia structure and released spores afterwards (**Figure 3B**; **Supplementary Figure S4B**). These observations suggest that HDAC-depended on histone acetylation state remodeling is intimately required in the full course of developmental stages in response to wound stress. Even after cell fate transition was initiated after wounding, HDAC activity was still required in regulating cell cycle progress and releasing of spores. Moreover, the appropriate transcriptional regulation by HDAC on the first day after wounding was fundamental to triggering cell fate transition, as evidenced by the significantly dropped probability of spore formation when they disturbed by transient SAHA treatment on day 1.

### 3.4 SAHA-treatments changed the transcriptional responses to wound

**Figure 4:**
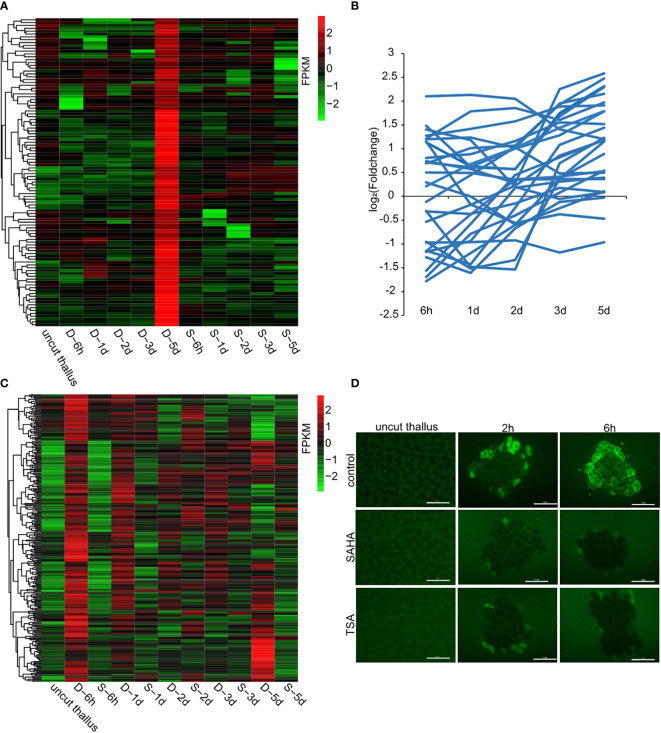
Candidate genes that were transcriptionally regulated by HDACs. **(A)** Transcriptional levels of sporangia-specific genes at different time points in control media (marked as D-) and SAHA-supplemented media (marked as S-). The control sample stands for uncut thalli. The log_2_ value of FPKM foldchange compared to the control sample at each time point for individual genes are displayed, as indicated by the color bar. **(B)** Transcriptional foldchanges of genes related to DNA replication in SAHA-supplemented media. **(C)** Heatmap showing transcriptional levels of the 291 candidate genes in control media and SAHA-supplemented media. The D- and S- samples at the same time point placed adjacently to underline the contrast between them; **(D)** ROS signals in control media, SAHA-supplemented media and TSA-supplemented media after wounding (Bars = 50 μm).

As a way to investigate the regulatory mechanism of HDACs in wound-induced cell reprogramming in *P. yezoensis*, we collected the global gene-expression profiles of fragments in SAHA-supplemented media during spore formation and compared them with those in control media that we published previously (**Supplementary Table S1**). We chose eight genes from the transcriptomic data and detected their transcriptional levels using qPCR. The two methods gave relatively consistent trends of variation for either control or SAHA-treated samples (**Supplementary Table S2**, **Supplementary Figure S5**). We first analyzed the transcriptional levels of sporangia-specific genes which were previously found specially expressed in sporangia and pre-spores on the 5^th^ day in the control medium. In SAHA-treated samples, transcription of these genes was not only barely detected during the time course after wounding, but also remained inactive on the 5^th^ day (two of cellulase genes were experimentally verified in (**Supplementary Figure S5**), except only three that showed weak transcription at several time points (**Figure 4A**). Transcriptional “silence” of these gene markers of sporangia in SAHA-treated samples further confirmed the lack of cell fate transition from somatic cells to spores.

As the cell division was a typical event during WIS formation, we then investigated the transcriptional variations of related genes in SAHA-treated samples. Among the 28 genes involved in DNA replication, 7 were up-regulated and 7 were down-regulated at 6h, but none of them, except one, had a foldchange greater than 4. Afterwards, most of them exhibited weak transcriptional variations (**Figure 4B**). Apparently, the global trend of transcriptional variations under SAHA treatment was not synchronized as the one during WIS formation that showing a coordinated “down-regulation first and up-regulation at day 2” pattern. Synchronized transcription was also not found in the genes involved in mismatch repair (**Supplementary Figure S6**). This is consistent with our observation of a lack of cell division events.

Compared with transcriptional levels at certain time points in control media, the most abundant of genes exhibited varied regulation at 6h after wounding under SAHA treatment. Combining of our finding that HDAC-regulated transcriptional response in the 1^st^ day is vital to determine whether cell fate transition would be initiated, we therefore focused on the transcriptional variations of genes at 6h. Generally, histone acetylation is associated with gene activation and inhibiting HDAC activities will promote gene expression. To identify target candidates of HDACs, we first investigated genes up-regulated under SAHA-treatment albeit down-regulated under control media. 194 genes were identified. The functional categories they encoded were mainly metabolic pathways, including amino acid metabolism, cofactor metabolism, lipid metabolism, et al. (**Supplementary Figure S7A**). Interestingly, we found one gene encoding the pyruvate decarboxylase (PDC) and one encoding aldehyde dehydrogenase (ALDH). PDC and ALDH were key enzymes mediating biosynthesis of acetate and their genomic loci were directly bound and transcriptionally regulated by HDA6 in response to drought stress in *Arabidopsis* (Kim et al., 2017). The two genes were up-regulated by more than two folds at 6h and 1d under SAHA-treatment, indicating a possible epigenetic control of acetate accumulation by histone acetylation in *P. yezoensis* (**Supplementary Figures S8A, B**). Seven genes encoded transcriptional factors or regulators, including two C2H2-, one bZIP- and one bHLH-related transcription factors as well as three helicase-domain-containing chromatin remodeling proteins.

To further identify genes that were associated with wound-induced cell fate transition and potentially regulated by HDACs, we selected genes that meet the following three criteria: a, exhibited significant transcriptional up-regulation at 6h in control medium; b, exhibited weak variation, or down-regulation in SAHA-treated medium; c, orthologs in *Pyropia haitanensis* also exhibited weak variation or down-regulation at 6h after wounding (Guan et al., 2022). We identified 291 genes in this manner (**Figure 4C**; **Supplementary Table S3**). Functional categories of ribosome biogenesis, transcription, RNA processing and transport were the top 3 KEGG pathways that were enriched (**Supplementary Figure S7B**). 10 of these genes encoded transcriptional factors or regulators, including three MYB-related and two C2H2-related transcription factors. Interestingly, two genes involved in SWI/SNF chromatin remodeling complex and a GNAT-type histone acetyltransferase were also included, indicating the crosslink in histone acetylation components. Additionally, there’re 8 genes encoding serine/threonine protein kinase. Six of them had their *P. haitanensis* orthologs exhibited weak transcriptional variation or significant downregulation, indicating that they were tightly associated with wound-induced cell fate transition in *P. yezoensis*. One of the protein kinases harbored EF_hand motifs and was annotated to be calcium-dependent kinase. Two genes encoding calmodulin (CaM) and glutamate receptor (GLR) were also included, strengthening the essential role of Ca^2+^ signaling in stimulating spore formation (**Supplementary Table S3**, **Supplementary Figure S9**). ROS was found to be another signal molecule in systematic wound signaling. We found 3 genes related to ROS biogenesis in this list, including two RBOH genes and one galactose oxidase gene (**Supplementary Table S3**, **Supplementary Figure S9**). To further investigate the regulation of ROS signaling by HDACs, we detected the ROS accumulation in thallus cut fragments under SAHA treatment. Unlike the gradual propagation from wound sites to internal intact cells in control cut fragments, in SAHA-treated cut fragments, we observed ROS signals in cells facing cut edges by 1-2 h post-wounding while the internal cells remained blank. At 6h-12h, ROS signals were barely be detected (**Figure 4D**). Similar suppression of the ROS signal was observed when treated by TSA.

## 4 Discussion

Somatic cells in *P. yezoensis* thalli experience dedifferentiation and reprogram into asexual spores when they are under wound stress (Chen et al., 2020). These complex physiological processes are associated with dynamic transcriptional responses (Guan et al., 2022). However, little is known about the molecular mechanism that directs the wound-induced gene expression changes and subsequent cellular reprogramming. Previously researchers performed comparative evolutionary and gene expression analyses of core subunits of the SWI/SNF chromatin-remodeling complex in red algae, implicating the association of reforming chromatin structure in modulating gene expressions (Stiller et al., 2018). Although it acts as one of the main canonical mechanisms in chromatin remodeling, histone acetylation was not investigated afterwards in red algae. With the availability of two *Pyropia* genomes (Cao et al., 2020; Wang et al., 2020), we found four and nine genes encoding histone acetyltransferase and histone deacetylase, respectively, in *P. yezoensis* and showed high sequence similarities with their individual orthologs in *P. haitanensis* (Data not shown). This study is the first investigation of histone -acetylation-mediated epigenetic regulation of wound-induced spore formation in *P. yezoensis* and sheds new light on the transcriptional regulatory mechanisms in response to wound stress in red algae.

Several transcription factor genes have been identified to be targets of histone acetylation in response to abiotic and biotic stresses in plants. In somatic embryogenesis of Arabidopsis explants, histone H3 and H4 acetylation control the expression of some critical transcription factor genes, including *LEC1*, *LEC2* (*LEAFY COTYLEDON 1, 2*), *FUS3* (*FUSCA 3*) and *MYB118* (*MYB DOMAIN PROTEIN 118*) (Morończyk et al., 2022). All of these TF genes are confirmed to be of vital roles in somatic embryogenesis. *LEC1* encodes the HAP3 subunit of the CCAAT box-binding factor. A *P. yezoensis* gene py08401 met the mutual-best-hits criterion and might be its ortholog. The transcription level of py08401 exhibited slight downregulation after wounding whenever there’s SAHA treatment (**Supplementary Figure S8C**). Both LEC2 and FUS3 are plant-specific families of TFs with a highly conserved B3 domain albeit lack of homologs in *P. yezoensis*. There’re 23 MYBs or MYB-related family transcription factor genes in *P. yezoensis*, but none is phylogenetically closed to plant MYB 118. Overall, since plants and red algae are two highly divergent lineages, regulatory targets of histone acetylation identified in plants are less likely to be the same in red algae. In this study, we found MYB-related and C2H2 TFs were promising targeted genes of histone acetylation in promoting wound-induced cell reprogramming. Since these five genes are also evolutionarily conserved in *Gracilariopsis* and *Chondrus* genomes (Lee et al., 2018), it’s possible that the transcriptional regulatory network they constitute with HDAC is shared in red algae.

Despite being potentially harmful to the cell, increasing evidence suggest that ROS are crucial signaling molecules in regulating plant growth and development, as well as response to diverse biotic and abiotic stresses. Long-distance transmission of ROS (also called systematic signaling) was observed in wounded leaves and confirmed to be essential in triggering cell regeneration in Arabidopsis. Recent studies have revealed complex links between ROS signaling and histone acetylation. Different levels of ROS caused by diverse developmental or environmental signals regulate the expression of HDACs and HATs and reform histone acetylation status that either mitigate or mediate the plant growth and stress response by changing downstream gene transcription (R et al., 2020). Conversely, there’re wealth of information showing that altering histone acetylation status affects ROS generation. Inhibiting HDAC activity in corn roots led to a continuous rise in intracellular ROS concentration, and caused cell cycle arrest at preprophase in the root meristem zones (Zhang et al., 2017). Overexpression of the HAT gene (TaHAG1) triggers increased H3 acetylation and transcriptional upregulation of genes responsible for hydrogen peroxide production under salt stress, leading to improved salt tolerance in polyploidy wheat (Zheng et al., 2021). In *Pyropia*, the vital role of ROS as signal molecules in response to abiotic stress has been uncovered in previous studies. In the wound-induced spore formation, an increased expressional level of RBOH genes was observed immediately after wounding, accompanied by gradual propagation of ROS from wound sites to other intact cells (Guan et al., 2022), indicating the association of ROS systematic pathways in triggering wound-induced spore formation. Meanwhile, inhibiting ROS generation *via* DPI, the chemical inhibitor of RBOH, strongly repressed the release of asexual archeospores in *P. yezoensis* (Gui et al., 2022). However, little is known about the regulation of ROS generation. In this study, we further found that inhibiting HDAC activity by SAHA arrested the up-regulation of RBOH, and ROS propagation in cut fragments was attenuated, leading to failure in starting cell fate reprogramming. Taken together, our findings demonstrate that ROS production mediated by histone deacetylation is a positive signal molecule to modulate the fate transition of somatic cells in *P. yezoensis* thalli under wounding stress.

## Data availability statement

The datasets presented in this study can be found in online repositories. The names of the repository/repositories and accession number(s) can be found in the article/**Supplementary Material**.

## Author contributions

DW designed the research. XG, HQ and WQ performed research. SS, YP, XZ and NC prepared samples. YM and AP contributed in funding and paper editing. DW analyzed data and wrote the paper. All authors contributed to the article and approved the submitted version.

## Supplementary material

Supplementary Table 1 Statistics of Transcriptome sequencing data.

Supplementary Table 2 Primer sequences used in qPCR.

Supplementary Table 3 Functional annotation and transcriptional levels of SAHA-responsive genes. Genes with repressed transcription by SAHA were listed in sheet 1 and genes with activated transcription by SAHA was in sheet 2.
