# Supplementary material for: Histone acetylation functions in the wound-induced spore formation in nori
Source: Front Plant Sci. 2022 Dec 8;13:1064300. doi: 10.3389/fpls.2022.1064300 (PMC9773553; doi:10.3389/fpls.2022.1064300)
Supplement: Supplementary file 1 [file DataSheet_1.pdf]

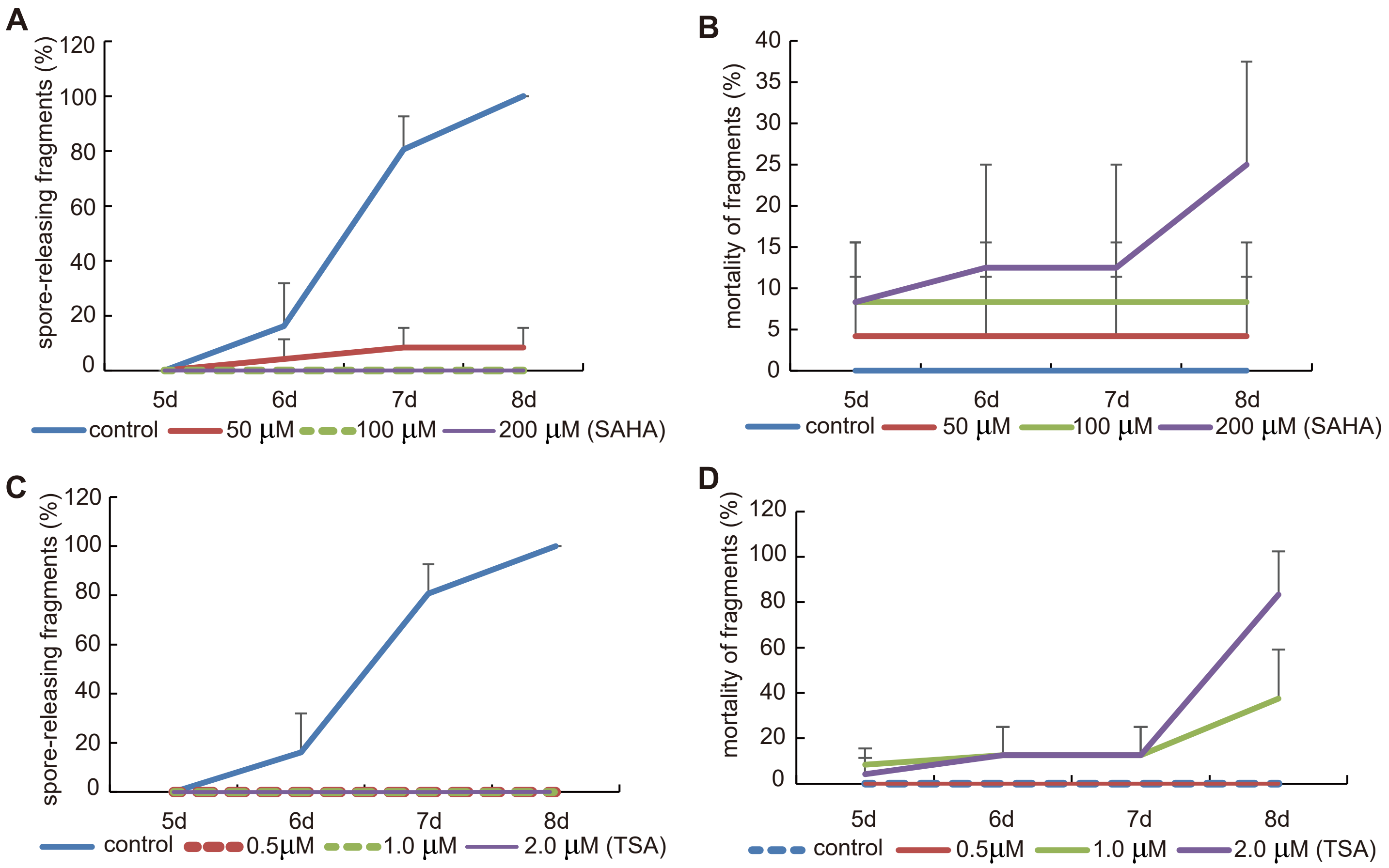

Figure S1. Effects of SAHA and TSA at different concentrations to WIS formation and cell mortality. (A) The percentage of spore-releasing fragments under different concentrations of SAHA. Error bars represent the SD (n = 3, 30 fragments/replicate as described in "Materials and Methods" section). (B) The mortality of fragments under different concentrations of SAHA. (C) The percentage of spore-releasing fragments under different concentrations of TSA. (D) The mortality of fragments under different concentrations of TSA.

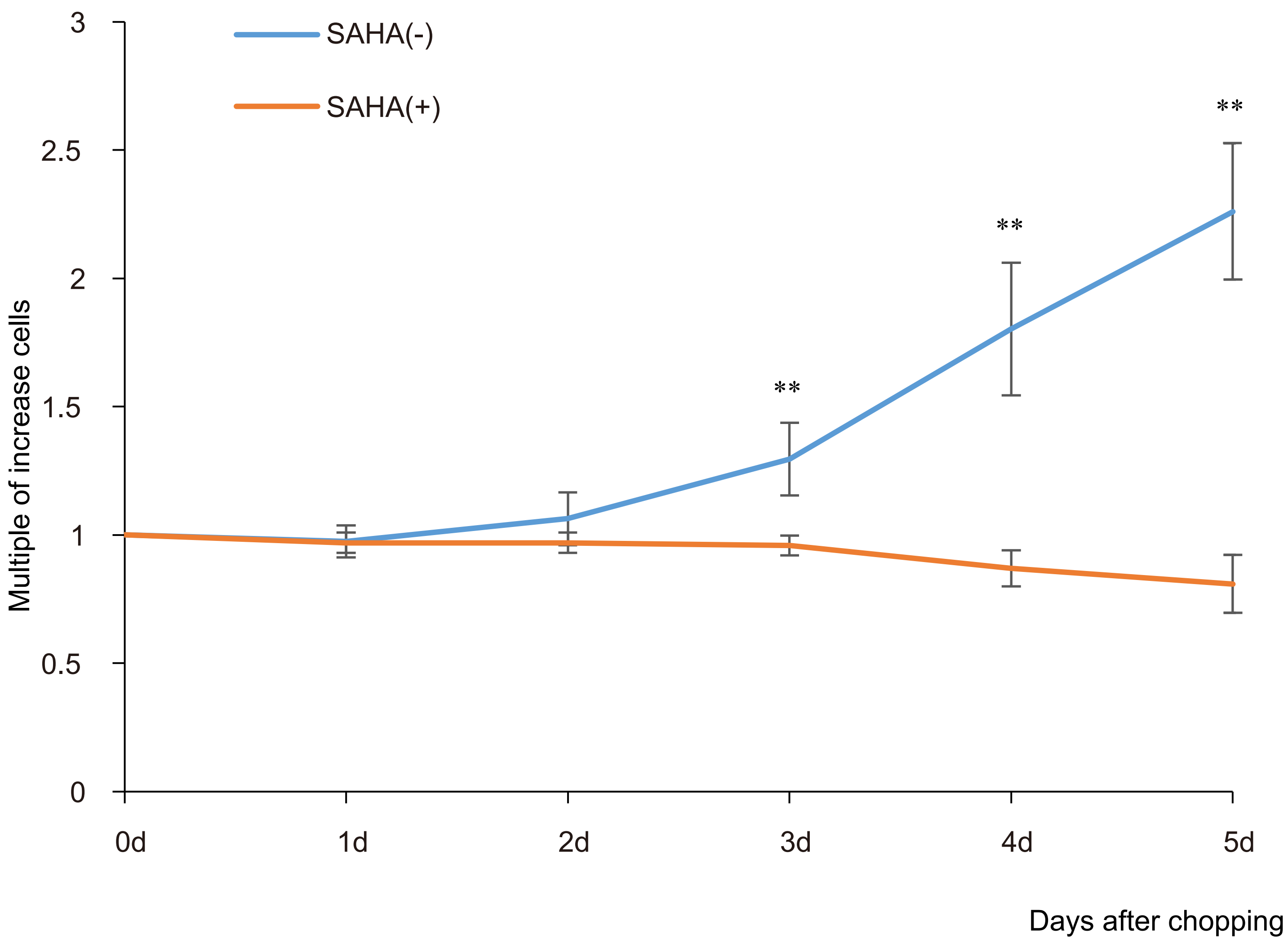

Figure S2. Average cell numbers in each cut fragment along the time course in control and SAHA-supplemented media. Y-axis stands for the ratio to the original cell number (0h after wounding) in cut fragment.

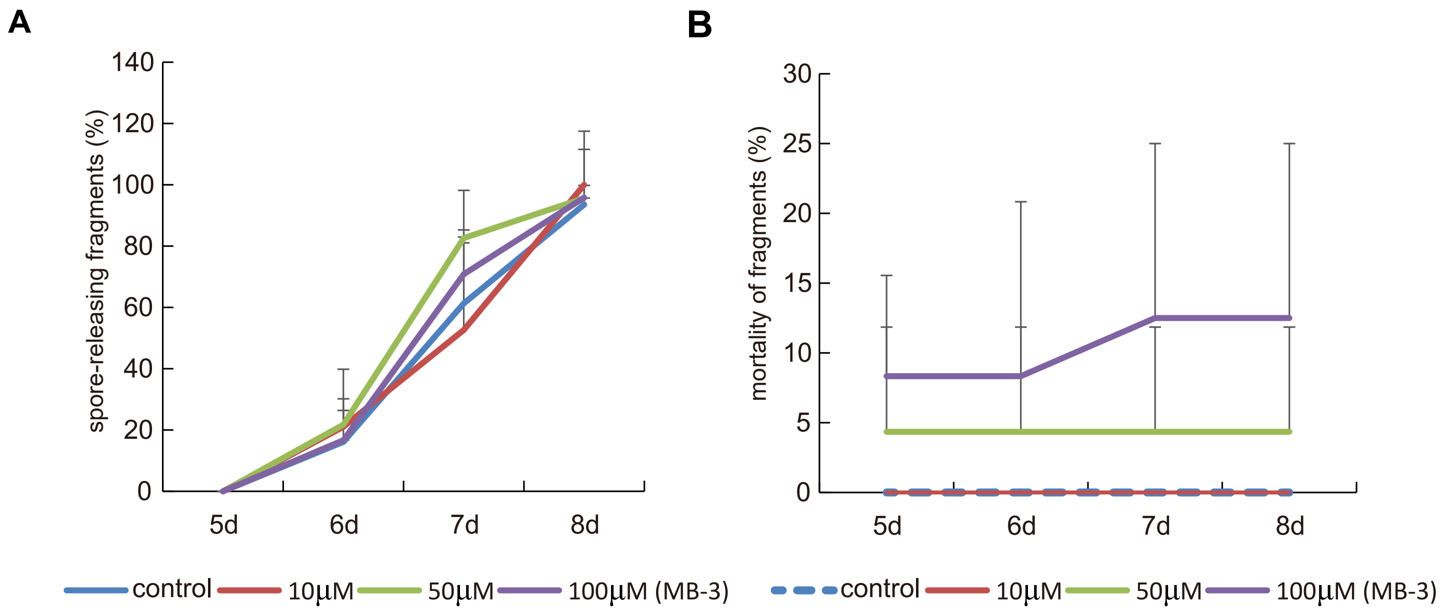

Figure S3. Effects of MB-3 at different concentrations to WIS formation (A) and cell mortality (B). Error bars represent the SD (n = 3, 30 fragments/replicate).

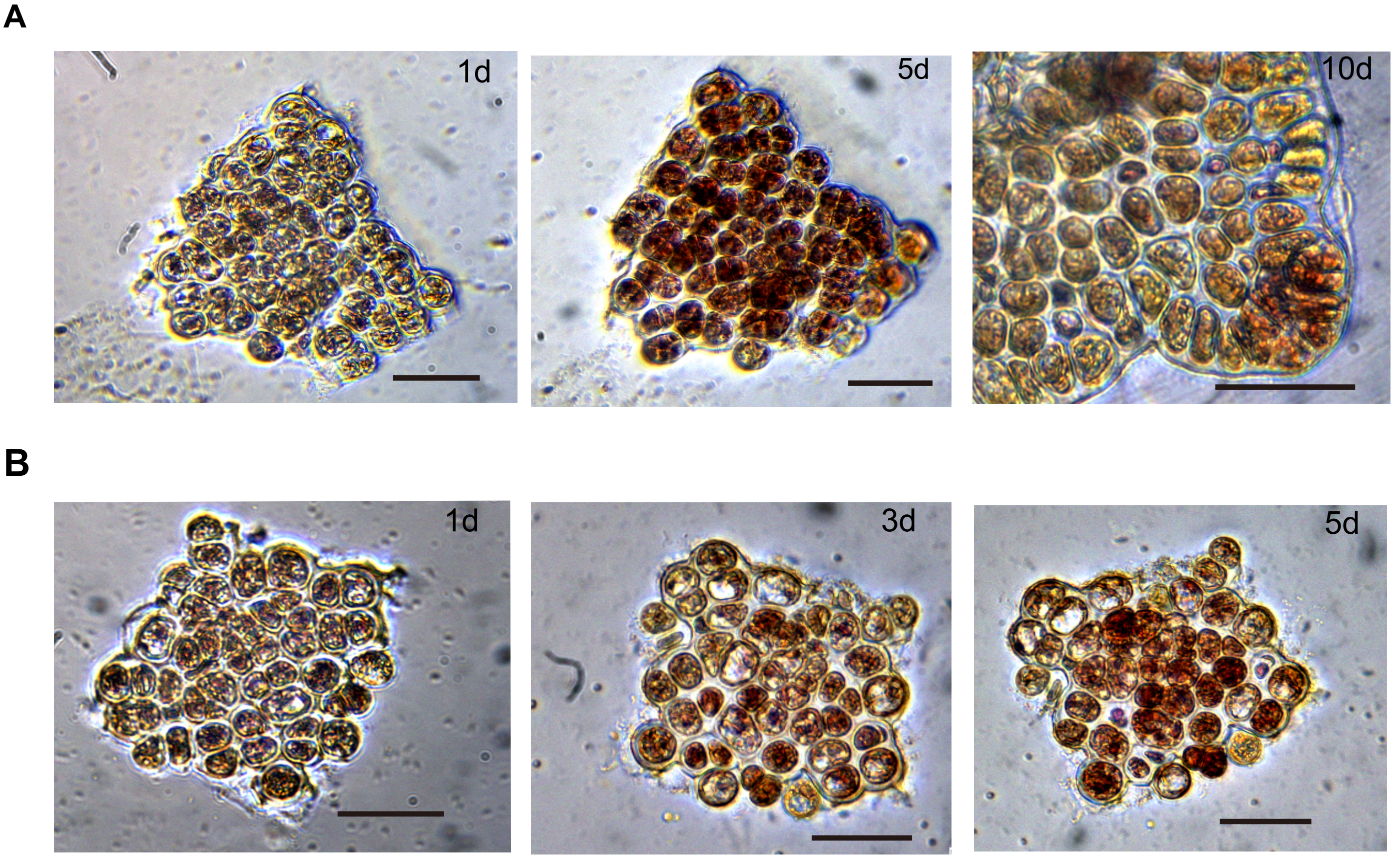

Figure S4. The morphological changes of 5d-SAHA-added (A) and 5d-SAHA-removed (B) thallus fragments. Bar = 50 μm

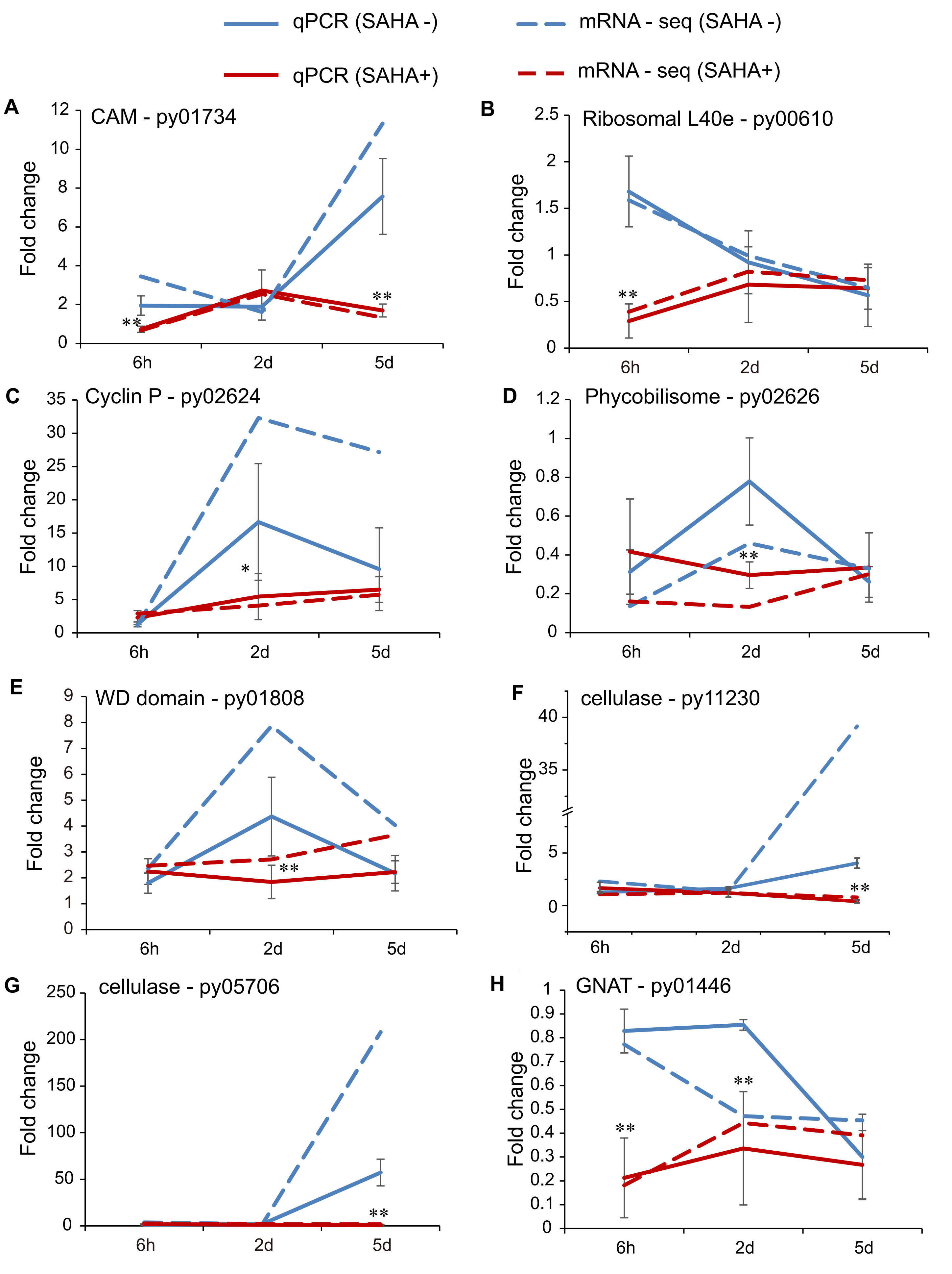

Figure S5. Transcript dynamics of *P. yezoensis* genes related to this study as measured by mRNA-seq (dash line) and qRT-PCR (solid line). *, 0.01 < P -value < 0.05, **, P -value < 0.01.

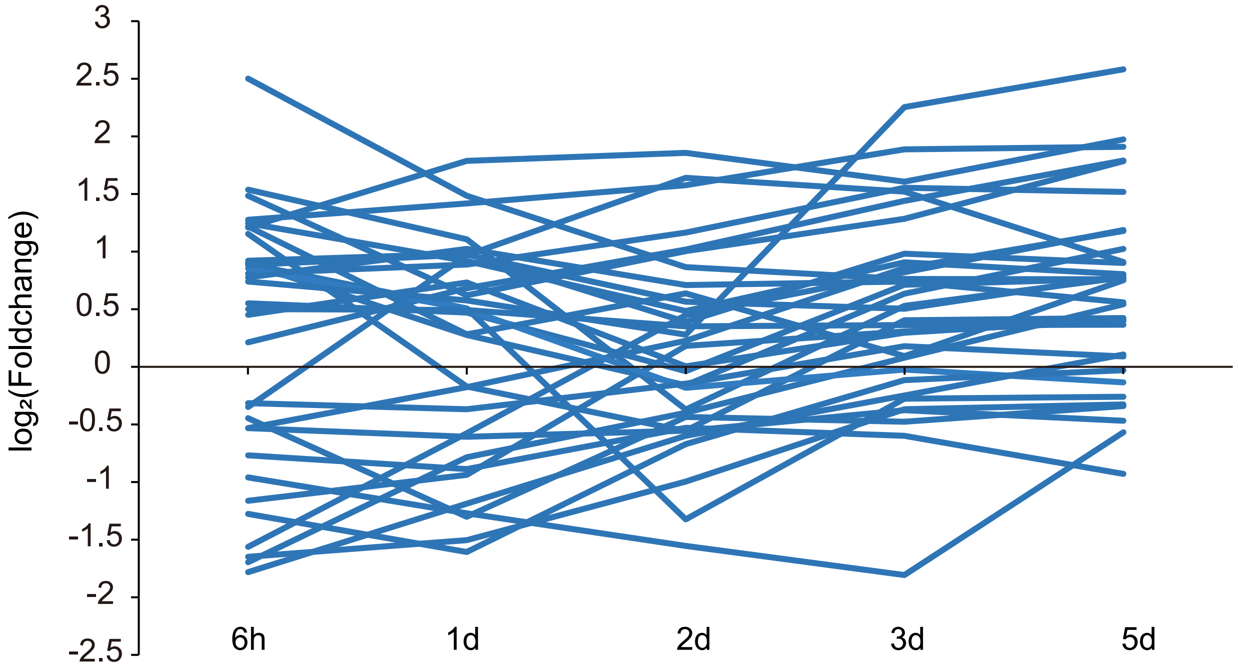

Figure S6. Transcription variation of mismatch repair and base excision repair related genes in SAHA-supplemented media.

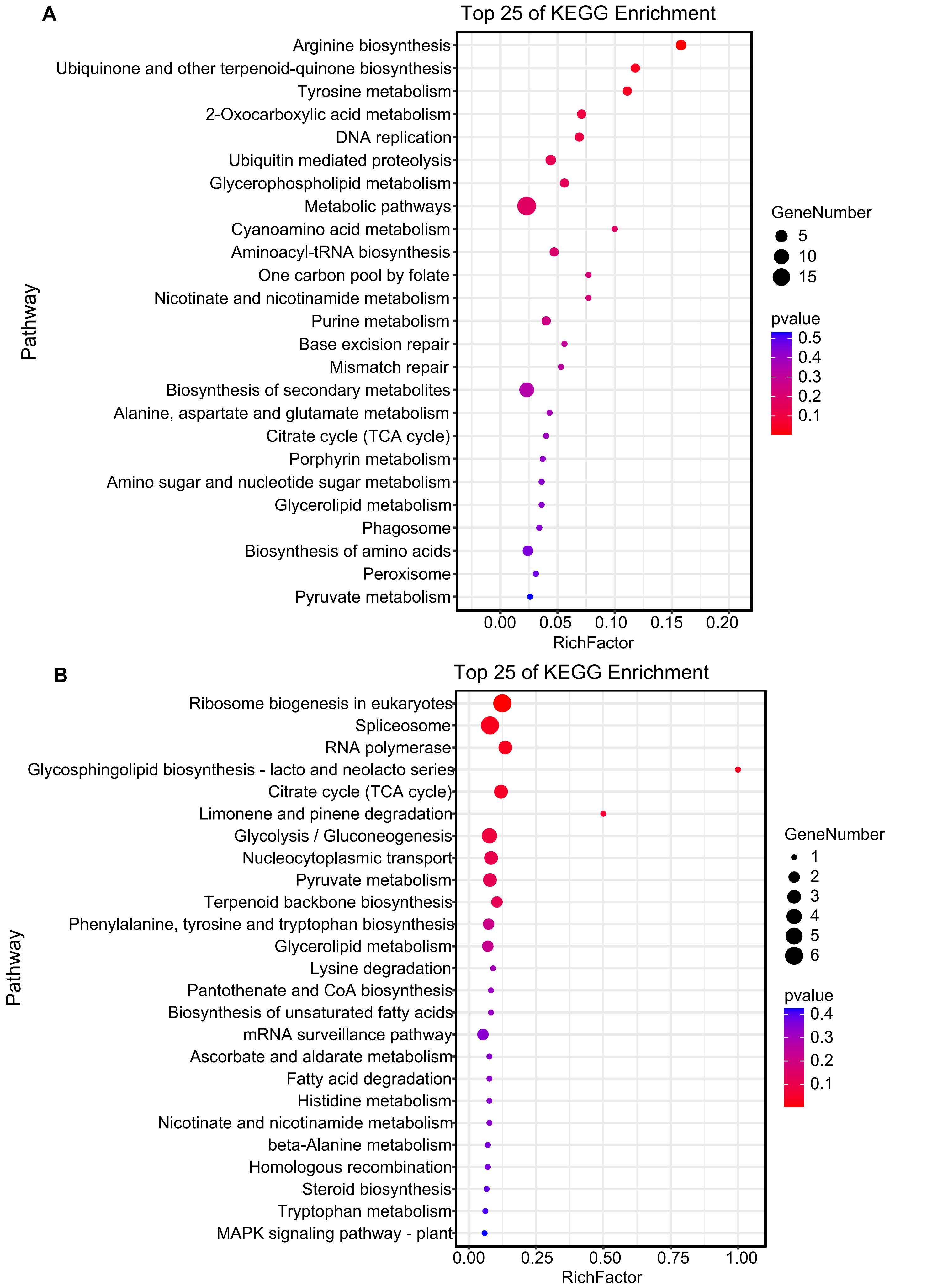

Figure S7. KEGG enrichment pathway analysis of SAHA-responsive genes. (A) Enriched functional categories encoded by the 194 genes with activated transcription by SAHA. (B) Enriched functional categories encoded by the 291 genes with repressed transcription by SAHA.

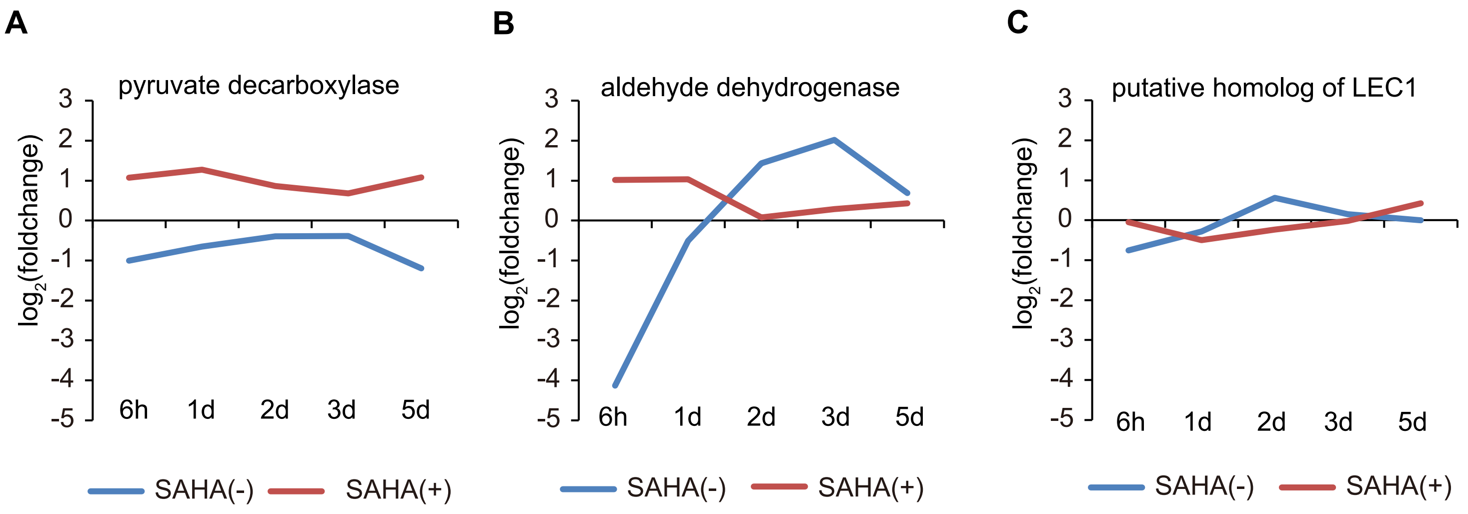

Figure S8. Transcriptional variations of genes encoding PDC (A), ALDH (B) and the putative homolog of LEC1 in *P. yezoensis* cut fragments in control or SAHA-supplemented media.

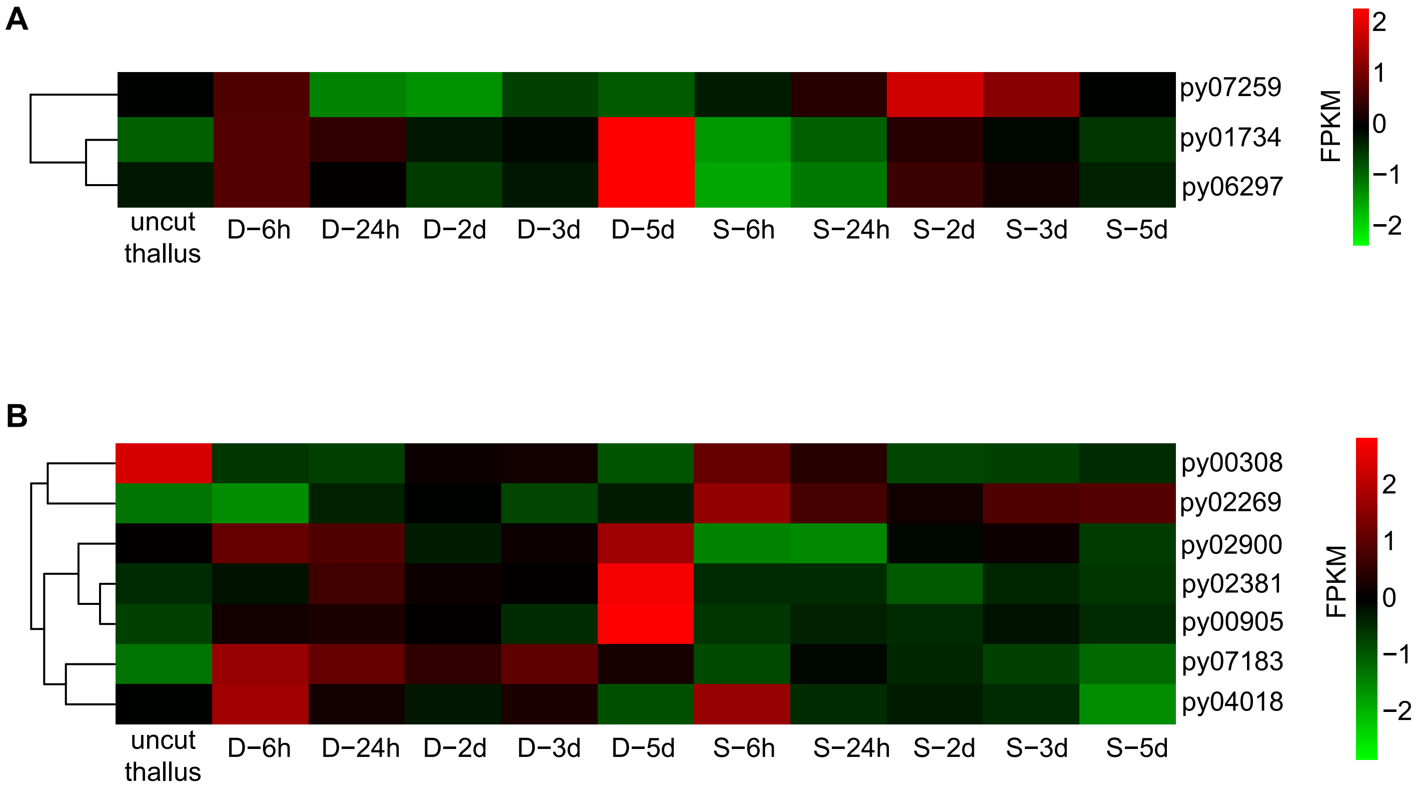

Figure S9. Heatmap showing the transcriptional levels of CaM(A) and RBOH(B) genes along the time course in control and SAHA-supplemented media.
