# Supplementary material for: Histone acetylation functions in the wound-induced spore formation in nori
Source: Front Plant Sci. 2022 Dec 8;13:1064300. doi: 10.3389/fpls.2022.1064300 (PMC9773553; doi:10.3389/fpls.2022.1064300)
Supplement: Supplementary Table 2 — Primer sequences used in qPCR. [file Table_2.docx]

| gene ID | gene name | Forward primer | Reverse primer |
| --- | --- | --- | --- |
| py10446 | GNAT | 5' GCCGCACCATTGGCTACCG 3' | 5' CCTCAATGTGGCCGGCTG 3' |
| py01734 | CAM | 5' CCTCACCCTCATGTCACGCAAG 3' | 5' CCCGGATCATCTCGCTCACC 3' |
| py00610 | Ribosomal L40e | 5' TGTGATTGAGCCCACCCT 3' | 5' TCTTCTTGCGGCAGTTGGTC 3' |
| py02624 | Cyclin P | 5' GTGTCGCCAAGAAGGCGAG 3' | 5' GGTGAGAAGGCCGCTAAGG 3' |
| py01808 | WD | 5' AGCGCCGTCTTCTCACCCAAC 3' | 5' CCCGCGTACGTCTTGACCACC 3' |
| py02626 | Phycobilisome | 5' CGCTCTGGTTAAGTCCAACGA 3' | 5' TCCATGTACGCCTCATAGCC 3' |
| py11230 | cellulase1 | 5' CAGTGGCGTGTATGGGGA 3' | 5' CGGTCGGGCTGGTTGA 3' |
| py05706 | cellulase2 | 5' CGAGACCGCCCTTGTCCAC 3' | 5' CGTCTCCATTGTACGCAGTGACC 3' |
| py04835 | UBC | 5' CGCTGACCGTTTCCAAG 3' | 5' CGACTGCGGTTGGACTT 3' |
